# Transplacental induction of fatty acid oxidation in term fetal pigs by the peroxisome proliferator-activated receptor alpha agonist clofibrate

**DOI:** 10.1186/s40104-015-0010-7

**Published:** 2015-03-26

**Authors:** Xi Lin, Sheila Jacobi, Jack Odle

**Affiliations:** Laboratory of Developmental Nutrition, Department of Animal Sciences, North Carolina State University, Box 7621, Raleigh, NC 27695 USA

**Keywords:** Clofibrate, Fatty acid oxidation, Pigs, Placenta transfer

## Abstract

**Background:**

To induce peroxisomal proliferator-activated receptor α (PPARα) expression and increase milk fat utilization in pigs at birth, the effect of maternal feeding of the PPARα agonist, clofibrate (2-(4-chlorophenoxy)-2-methyl-propanoic acid, ethyl ester), on fatty acid oxidation was examined at full-term delivery (0 h) and 24 h after delivery in this study. Each group of pigs (n = 10) was delivered from pregnant sows fed a commercial diet with or without 0.8% clofibrate for the last 7 d of gestation. Blood samples were collected from the utero-ovarian artery of the sows and the umbilical cords of the pigs as they were removed from the sows by C-section on day 113 of gestation.

**Results:**

HPLC analysis identified that clofibric acid was present in the plasma of the clofibrate-fed sow (~4.2 μg/mL) and its offspring (~1.5 μg/mL). Furthermore, the maternal-fed clofibrate had no impact on the liver weight of the pigs at 0 h and 24 h, but hepatic fatty acid oxidation examined in fresh homogenates showed that clofibrate increased (*P* < 0.01) ^14^C-accumulation in CO_2_ and acid soluble products 2.9-fold from [1-^14^C]-oleic acid and 1.6-fold from [1-^14^C]-lignoceric acid respectively. Correspondingly, clofibrate increased fetal hepatic carnitine palmitoyltransferase (CPT) and acyl-CoA oxidase (ACO) activities by 36% and 42% over controls (*P* < 0.036). The mRNA abundance of CPT I was 20-fold higher in pigs exposed to clofibrate (*P* < 0.0001) but no differences were detected for ACO and PPARα mRNA between the two groups.

**Conclusion:**

These data demonstrate that dietary clofibrate is absorbed by the sow, crosses the placental membrane, and enters fetal circulation to induce hepatic fatty acid oxidation by increasing the CPT and ACO activities of the newborn.

## Background

High postnatal mortality has been recognized as a critical problem by the modern swine industry since the 1990’s. Although genetic improvement has increased the number of pigs born alive per litter, the death rate of newborn pigs during the postnatal period has increased slightly in recent years, from 13.2% in 2006 [1] to 14.8% in 2010 [2]. The epidemiology of postnatal mortality is complex, but data clearly show that inadequate energy (starvation) ranks among the leading causes, especially in the first three days after birth. The immediate postnatal period poses the greatest challenge to the energy balance of neonates, which must rapidly switch from carbohydrates supplied by the mother in utero [3] to predominantly lipids supplied via milk. Sow milk fat concentration is about 6% at birth and increases to 10% within 24 h. This is 67–70% higher than human colostrum (3.5–6%) and constitutes up to 60% of the energy in the milk, indicating that the milk fat is the primary energy source for newborn pigs after birth. Thus, it is critical for newborn pigs to use milk fat efficiently at birth to survive. However, results from previous studies show that over 85% of the fatty acids taken up by newborn pig hepatocytes are re-esterified but not oxidized [4]. In addition, 80% of the acetyl-CoA produced from fatty acid β-oxidation in mitochondria and/or peroxisomes is converted to acetate rather than ketone bodies [5]. These results indicate that newborn pigs have a limited capacity to oxidize milk fat for energy production at birth.

Hepatic fatty acid β-oxidation pathways are controlled mainly by the key enzymes carnitine palmitoyltransferase I (CPT I) in mitochondria and acyl-CoA oxidase (ACO) in peroxisomes. The activities of these key enzymes are transcriptionally regulated by the peroxisomal proliferator-activated receptor α (PPARα; [6]). This central transcription factor, PPARα, can be activated by its natural ligands such as eicosanoids and long-chain fatty acids or a host of pharmaceutical agonists including fibrates, a class of amphipathic carboxylic acids. Clofibrate, a potent pharmaceutical PPARα agonist of the fibrate class, stimulates hepatic mitochondrial and peroxisomal fatty acid β-oxidation by inducing PPARα target genes such as CPT I and ACO mRNA expressions and protein activities. The induction of CPT I and ACO by clofibrate via activation of PPARα has been documented in rodent species [7], laying hens [8], dairy cows [9] and pigs [10,11]. Data from our previous studies showed that the total CPT and ACO activity increase 2- and 3-fold respectively in the livers of pigs fed clofibrate for 2 weeks. Feeding clofibrate also increases hepatic peroxisomal and mitochondrial β-oxidation of [1-^14^C]-palmitate by 60% and 186%, respectively, in addition to the increased CPT I and ACO specific activities [10]. Moreover, the increase in hepatic fatty acid oxidation is not accompanied by a significant hyperplasia or hepatomegaly [12] as observed in rodent species. These results indicate that the fatty acid oxidative capacity of pigs could be promoted via induction of PPARα without substantial peroxisome proliferation.

Recently we have demonstrated that supplementation of clofibrate could improve *in vivo* fatty acid oxidation in neonatal pigs [13]. However, increasing the fatty acid oxidative capacity of pigs at birth appears to be the key to improving energy utilization and increasing survivability. The question is whether the activities of CPT I and ACO can be increased by activation of PPARα prenatally. Whether PPARα agonist can be delivered to the newborn pigs via maternal diet is not known. Research concerning the impact of maternal feeding of clofibrate during pregnancy on fetal fatty acid oxidative capacity at birth or/and development after birth is very limited. Maternal dietary clofibrate induced peroxisomal proliferation in the liver and intestine tissues, and induced enterocyte peroxisomal catalase and peroxisomal bifunctional enzyme activities of fetuses in rats [14]. Maternal clofibrate also amplified fetal liver endoplasmic reticulum and peroxisomes, and increased the concentrations of peroxisomal membrane protein 70, the specific activity of dihydroxyacetone phosphate acyltransferase and catalase in the livers of fetal mice [15]. The relative expression of cytochrome P4504A mRNA was increased in the maternal liver and fetal rat tissues [16]. Furthermore, the increase in relative mRNA of ACO, CPT I, medium- and long-chain acyl-CoA dehydrogenases in the liver were also observed in fetal rats from the clofibrate-treated maternal rats [17]. These findings demonstrate that clofibrate is capable of crossing the placenta and increasing peroxisome proliferation and modulating specific gene expression. Therefore, we hypothesize that similar placenta transfer may occur in pigs, although the physiology of extra embryonic membrane attachment in swine are different from rodents and other species. The placental transfer of clofibrate has not been studied directly by measuring clofibrate in the circulation system of the pregnant animals and their fetuses, and the effect of the induction in enzyme activities and mRNA expression on fatty acid oxidative capacity is not known.

To test our hypothesis, we investigated the effect of maternal supplementation of PPARα agonist clofibrate on the development of oxidative capacity of newborn pigs at birth and 24 h after birth. We confirmed that PPARα agonist clofibrate transfers across porcine placental tissues by measuring maternal and fetal plasma clofibrate concentrations and increases hepatic oxidative capacity by measuring hepatic fatty acid oxidation in the newborns using [1-^14^C]-fatty acid substrates.

## Methods

### Animals, treatments and sampling

All procedures were approved by the Institutional Animal Care and Use Committee of North Carolina State University (IACUC number: 07-001-A). Twenty newborn pigs from either control (n = 10, body weight = 1.37 ± 0.047 kg) or clofibrate fed (n = 10, body weight = 1.20 ± 0.024 kg) sows were used in this study. Pregnant multiparous crossbred sows were housed at the North Carolina State University Swine Education Unit and were fed a standard gestation diet (3,265 Kcal ME/kg) with or without supplementation of 0.8% colfibrate (w/w) from day 105 of gestation until day 113. The clofibrate was diluted into 10 mL of ethanol and pre-mixed with ~50 g of feed each day. Sows were fed the premixes (clofibrate or vehicle) together with ~1/3 of their daily feed allotment first, to ensure complete consumption, and then the remaining 2/3 allotment was provided. The sows were given 1.75 kg diets per day in total and water was supplied ad libitum. Term fetuses were delivered by caesarian section on d 113 of gestation and plasma was collected simultaneously from the utero-ovarian artery of swine and from the umbilical vein of each fetal pig.

All newborn pigs were weighed and euthanized by AVMA-approved electrocution at time 0 (term fetus, n = 6) or 24 h (n = 4) after delivery. The pigs sampled at 24 h were housed at 35°C in a specialized nursery facility [18] and remained un-fed. Liver samples were immediately homogenized in a buffer (220 mmol/L mannitol, 70 mmol/L sucrose, 2 mmol/L HEPES, and 0.1 mmol/L EDTA; pH 7.2 at 0°C) using a 7 mL glass Pyrex hand homogenizer with 3 complete top to bottom strokes. Fatty acid oxidation in the fresh homogenate was measured immediately using [1-^14^C]-fatty acids. Homogenate protein was determined using the biuret method [19]. Samples were also immediately frozen in liquid nitrogen and stored at −80°C for enzyme and mRNA assays.

### Plasma assay

Clofibrate (2-(4-Chlorophenoxy)-2-methylpropionic acid ethyl ester), clofibric acid (2-(4-chlorophenoxy)-2-methylpropionic acid), 4-chlorophenylacetic acid and its metabolites in the plasma from the sows and term fetuses were extracted using solid-phase extraction (SPE) procedures as described by Du et al. [20]. The extraction was conducted using SPEC.C18 extraction cartridges (Ansys Technologies, CA, and USA). The clofibrate, clofibric acid, and other lipophilic components were eluted with a solvent mixture of acetonitrile : water : formic acid (79% : 20% : 1%, v/v/v) and were analyzed using a Waters HPLC Empower system (Milford, MA. USA). The separation of clofibrate, clofibric acid, and their metabolites was performed on a BDS Hypersil C18 (5 μm, 150 mm × 46 mm) analytical column with BDS C18 (5 μm, 4 mm × 3.0 mm) guard column from Phenomenex (Torrance, CA. USA). The isocratic HPLC system was used and the pump flow rate was set at 1 mL/min. The sample injection volume was 20 μL and the compounds were detected at 230 nm using a photodiode array detector (Waters 996).

### Fatty acid oxidation

Hepatic fatty acid oxidations were measured in fresh liver homogenates from the pigs at 0 or 24 h without suckling after delivery using [1-^14^C]-oleic acid (C18:1, the most enriched fatty acid in pig milk) and [1-^14^C]-lignoceric acid (C24:0, primary oxidized in peroxisomes) as substrates. The measurements followed the same procedure as described previously by Lin et al. [5]. Specifically, liver homogenates (~40 mg) were incubated in 25 mL Erlenmeyer flasks with a final of volume of 2 mL of the reaction medium with or without addition of antimycin A (50 μmol/L) and rotenone (10 μmol/L) described as previously [13] for determining peroxisomal β-oxidation by inhibition of the electron transport chain of oxidative phosphorylation. The reaction was initiated by adding 2 μmol of either [1-^14^C]-C18:1 (4.2 MBq/mmol) or [1-^14^C]-C24:0 (1 MBq/mmol) and terminated by adding 0.5 mL HCLO_4_. The ^14^CO_2_ collected in ethanolamine and the ^14^C-acid soluble products (ASP) processed from the acid-killed medium were quantified using liquid scintillation counter (Beckman LS 6000IC. Fullerton, CA. USA). The rate of total β-oxidation and the rate of peroxisomal and mitochondrial β-oxidation were calculated as described by Yu et al. [11].

### Enzyme and mRNA analysis

Hepatic ACO activity was determined in liver homogenates using a spectrophotofluorometric assay as described by Walusimbi-kisitu and Harrison [21] with slight modifications. The homogenates were prepared in an ice cold buffer containing 250 mmol/L sucrose, 1 mmol/L EDTA and 1% ethanol. After preparation, the homogenate (0.6 ± 0.035 mg protein) was incubated in a dark room at 37°C in 0.5 mL (final vol.) of a medium with or without 35 μmol/L palmitoyl-CoA for 20 min. The incubations were stopped by adding 2 mL of borate buffer (0.1 mol/L, pH 10). The medium contained 60 mmol/L Tris–HCl, 50 μmol/L FAD, 170 μmol/L CoA, 1 μmol/L scopoletin and 6% of BSA. 200 μL of the incubated medium was transferred into a 96 well plate and measured in a BioTek reader (Synergy HT) with emission at 460 and excitation at 360 nm (BioTek Instruments, Inc., Winooski, VT). The standard curve was generated using H_2_O_2_ (30%, w/w) and 150 IU peroxidase under the same incubation conditions and measurements.

CPT activity was measured in liver homogenates using a radio-enzymatic method as described previously by Bremer et al. [22]. The homogenate (65 ± 3.8 mg protein) was pre-incubated with 80 μmol/L palmitoyl-CoA in a medium containing 75 mmol/L KCl, 50 mmol/L HEPES, 0.2 mmol/L EGTA, and 1% of fatty-acid-free BSA in the presence or absence of 2.5 μmol/L malonyl-CoA, and the incubation was conducted at 30°C in a final volume of 1 mL for 3 min [5]. The measurement of the enzyme activity was initiated with 1 μmol of (l-methyl-^3^H)-carnitine (54 KBq/μmol) and terminated after 6 min by the addition of 2 mL of 6% of HClO_4_ (vol/vol). The radioactivity in palmitoyl-carnitine, generated during the incubation was extracted with water-saturated butanol and quantified using liquid scintillation spectrometry (Beckman LS 6000IC, Fullerton, CA. USA).

Assay of mRNA was conducted using the methods described by Lin et al. [5]. Total RNA was extracted from the liver samples using guanidine isothiocynate and phenol (TRIzol Reagent, Sigma Chemical, St. Louis, MO. USA). The extracted RNA was quantified using a NanoDrop spectrophotometer (NanoDrop Technologies, Wilmington, DE. USA), and the integrity of the RNA was confirmed using gel electrophoresis with SYBR Safe TM DNA gel stain from Invitrogen Life Technologies (Carlsbad, CA. USA). The RNA (10 μg/50 μL) then was treated with TuboDNase (Ambion, Austin, TX) according to the manufacturer’s instructions for removal of genomic DNA and transcribed using the iScriptTM Select cDNA Synthesis Kit provided with oligo (dT) primer mix. (Bio-Rad Laboratories, Hercules, CA). The mRNA abundance of PPARα, CPT I (L isoform) and ACO were determined using the MyiQ Single Color Real-Time PCR Detection System (Bio-Rad Laboratories, CA. USA). The determination was performed in triplicate with primers designed from pig-specific sequences available via GenBank and ordered from Sigma Genosys (St Louis, MO, USA). Amplification efficiencies were verified to be similar for the endogenous control GAPDH and the measured genes. Reactions contained cDNA with 0.4 μmol/L each of reverse and forward primers. The assay conditions and data calculations were the same as described previously [5].

### Chemicals

Clofibrate was purchased from Cayman Chemical Company (Ann Arbor, MI. USA). [1-^14^C]-oleic acid (C18:1) and [1-^14^C]-lignoceric acid (C24:0) were purchased from American Radiolabeled Chemicals, Inc. (St. Louis, MO. USA). 2-(4-Chlorophenoxy)-2-methylpropionic acid ethyl ester, 2-(4-chlorophenoxy)-2-methylpropionic acid, 4-chlorophenylacetic acid, peroxidase and all other chemicals were obtained from Sigma-Aldrich (St. Louis, MO. USA).

### Statistics

Data from fatty acid oxidation measurements were analyzed according to a split-plot design with the main plots in a completely randomized design using the SAS GLM procedure. The clofibrate effect was assigned to the main plot and pig postnatal age and fatty acid chain length effects were treated as the subplots. Least squares means ± SEM for variables are presented in tables and figures. Data from enzyme activity and mRNA enrichment assays were analyzed as a 2 × 2 factorial design using the GLM procedure. Least square means for treatment (clofibrate vs. control) and the postnatal age (0 vs. 24 h) effects were calculated. Differences between least squares means were determined using a Tukey test and considered significant when *P* ≤ 0.05.

## Results

### Plasma assay

**Figure 1:**
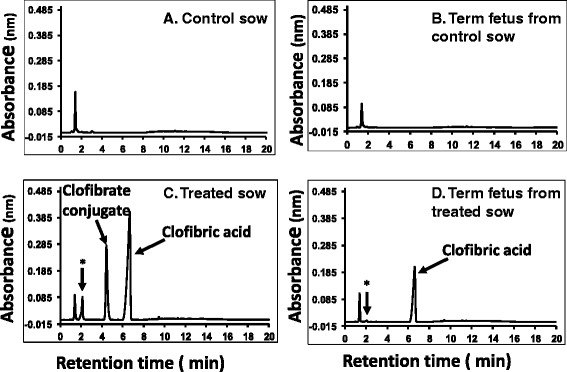
**Chromatogram of plasma samples from HPLC analysis. A.** control sow, **B.** newborn pigs from control sow, **C.** clofibrate-treated sow and **D.** term pigs from clofibrate-treated sow. See Methods for HPLC conditions and sample injection volumes. Compounds were identified by injection of available standards purchased from Sigma-Aldrich (St. Louis, MO) under the same HPLC conditions. *Unidentified peak.

Chromatograms from HPLC analysis confirmed that clofibric acid was present in the plasma of both the clofibrate-fed sow and their newborn pigs (Figure 1). In addition to clofibric acid, a clofibrate conjugate metabolite was detected in the plasma of the clofibrate-fed sow but not in the plasma of newborn pigs. An unidentified peak after the reagent peak was observed also in the chromatogram from the plasma analysis of the clofibrate-fed sow and the peak was tiny in the plasma of newborn pigs from the clofibrate-fed sow. No peaks of clofibric acid or its metabolites were detected in the plasma of control sows or their offspring.

### Fatty acid oxidation

**Figure 2:**
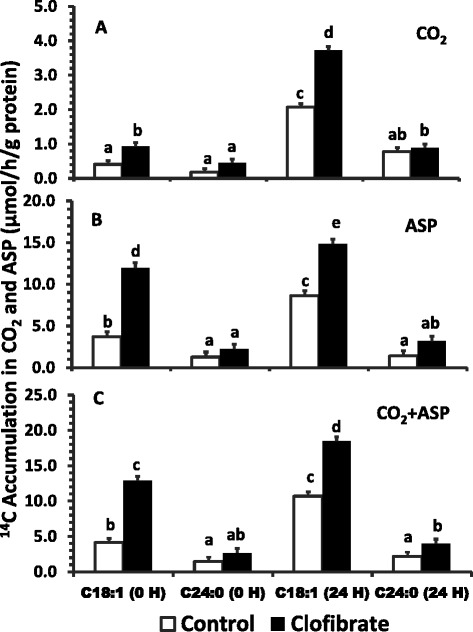
**The effect of maternal clofibrate on total (mitochondrial and peroxisomal) fatty acid oxidation in newborn pigs at 0 h and 24 h after delivery by C-section.** Total ^14^C labeled carbon accumulated in CO_2_ **(A)**, acid soluble products (ASP, **B**) and CO_2_ + ASP **(C)**. Values are least square means ± SEM (n = 6 for newborn pigs at 0 h and n = 4 for pigs at 24 h). ^abcde^ Bars within a panel lacking a common superscript differ (*P* < 0.05).

Results from total fatty acid oxidation in vitro (Figure 2) showed that clofibrate and postnatal age significantly stimulated hepatic β-oxidation of fatty acids (*P* < 0.0001), but the stimulatory effects of clofibrate and postnatal age for C18:1 oxidation were different from C24:0 oxidation. The ^14^C accumulations for C18:1 on average in CO_2_, ASP and CO_2_ + ASP (μmol/h/g protein) measured at 0 h and 24 h were 1.9, 2.2, and 2.1 fold higher, respectively, from clofibrate exposed pigs (2.32, 13.36 and 15.69) than control pigs (1.24, 6.17 and 7.41). Similarly, the ^14^C accumulations in CO_2_, ASP and CO_2_ + ASP (μmol/h/g protein) for C18:1 from both clofibrate treatment and control were on average 331%, 49% and 71% greater respectively in the livers of 24 h old pigs (2.89, 11.69 and 14.58) than the pigs at delivery (0.67, 7.83 and 8.51). In contrast with C18:1, the ^14^C accumulation for C24:0 on average in CO_2_ + ASP (μmol/h/g protein) measured at 0 h and 24 h was 1.8 fold higher from clofibrate exposed pigs (3.32) than control pigs (1.81), but no differences were detected in the ^14^C accumulation in CO_2_ and ASP (Figure 2, A & B). In addition, the ^14^C accumulation in CO_2_ from C24:0 was increased on average by 61% in the livers of 24 h old fasted pigs compared to the pigs at delivery (0.32), but there was no postnatal age effect on the ^14^C accumulations in ASP and CO_2_ + ASP (Figure 2, B & C). There was no interaction between clofibrate and pig postnatal age (*P* > 0.05).

**Figure 3:**
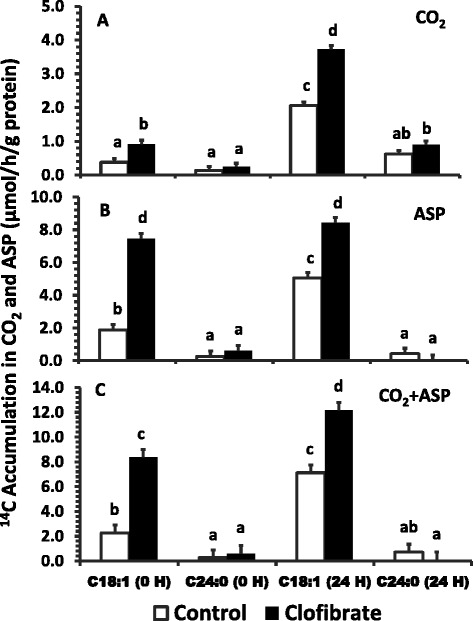
**The effect of maternal clofibrate on mitochondrial fatty acid oxidation in newborn pigs at 0 h and 24 h after delivery by C-section.** Total ^14^C labeled carbon accumulated in CO_2_ **(A)**, acid soluble products (ASP, **B**) and CO_2_ + ASP **(C)**. Values are least square means ± SEM (n = 6 for newborn pigs at 0 h and n = 4 for pigs at 24 h). ^abcd^ Bars within a panel lacking a common superscript differ (*P* < 0.05).

The mitochondrial oxidative flux of C18:1 to CO_2_ and ASP (Figure 3, A & B) as well as the total (CO_2_ + ASP, Figure 3, C) was affected by the treatment of clofibrate and the postnatal age (*P* < 0.0001). The ^14^C-accumulations in CO_2_, ASP and CO_2_ + ASP (μmol/h/g protein) in the livers of the pigs from sow received clofibrate (2.3, 13.1 and 15.5 respectively) were on average 2.1-fold higher than that from controls (1.2, 6.2 and 7.4 respectively). Similarly, the ^14^C-accumulations in CO_2_, ASP and CO_2_ + ASP also were increased on average by 3.1, 0.49 and 0.71 fold respectively in the pigs at 24 h after delivery (2.9, 11.7 and 14.6) as compared to the pigs at birth (0.7, 7.8 and 8.5). However, the stimulatory effect by clofibrate on the ^14^C-accumulations in ASP and CO_2_ + ASP tended to attenuate with the increase in postnatal age. There was no effect of age on the ^14^C accumulation in ASP in the pigs exposed to clofibrate (Figure 3B; *P* = 0.079). In contrast to C18:1, the mitochondrial oxidative flux of C24:0 to ASP and CO_2_ + ASP (μmol/h/g protein) remained similar in the livers of the pigs exposed to clofibrate (0.3, 0.83) and the controls (0.4, 0.9), although the flux to CO_2_ tended to be higher in the liver from the clofibrate-exposed pigs than control pigs. There was no significant change in the oxidative flux of C24:0 as the postnatal age increased (*P* > 0.05).

**Figure 4:**
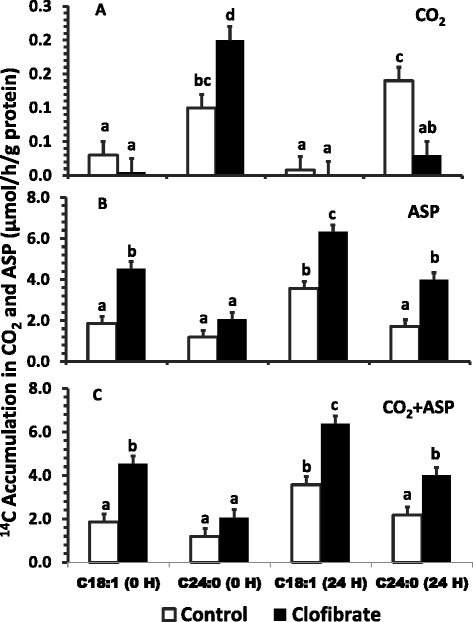
**The effect of maternal clofibrate on peroxisomal fatty acid oxidation in newborn pigs at 0 h and 24 h after delivery by C-section.** Total ^14^C labeled carbon accumulated in CO_2_ **(A)**, acid soluble products (ASP, **B**) and CO_2_ + ASP **(C)**. Values are least square means ± SEM (n = 6 for newborn pigs at 0 h and n = 4 for pigs at 24 h). ^abcd^ Bars within a panel lacking a common superscript differ (*P* < 0.05).

No substantial ^14^C-accumulation in CO_2_ (<0.25, μmol/h/g protein) was detected in peroxisomes (Figure 4, A). Clofibrate exposure and postnatal age had no impact on the negligible peroxisomal oxidative flux to CO_2_ from C18:1, but significantly increased the flux to ASP and CO_2_ + ASP (*P* < 0.0001; Figure 4, B & C). The ^14^C accumulation in the ASP and CO_2_ + ASP (μmol/h/g protein) from C18:1was on average 2 fold greater from clofibrate-treated (5.4, 5.5) than control (2.7, 2.7) pigs, and 56% higher from the 24 h-old pigs than that from pigs at delivery (1.3, 1.7). In comparison to C18:1, clofibrate increased the ^14^C accumulation from C24:0 peroxisomal oxidation in CO_2_ in the liver of pigs at delivery but decreased in the liver of 24 h-old pigs. Thus there was no significant difference (*P* = 0.93) in the average flux to CO_2_ (μmol/h/g protein) between clofibrate-treated (0.15) and control pigs (0.14). There were no effects of clofibrate on the ^14^C accumulation in ASP and CO_2_ + ASP from C24:0 in pigs at delivery, but the ^14^C accumulations was 84% greater from clofibrate-treated (4.0) than control in 24 h-old pigs (2.17). Postpartum age also had no influence on C24:0 oxidation in pigs at delivery (P > 0.05), but the ^14^C accumulation in ASP and CO_2_ + ASP was 94% higher from the clofibrate-treated pigs measured at 24 h (4.0) than at delivery (2.06).

**Figure 5:**
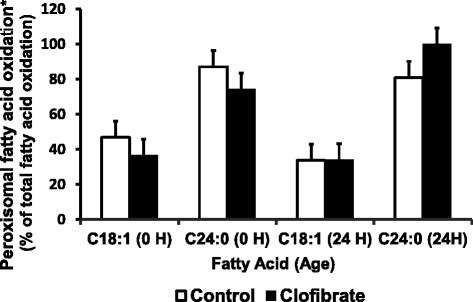
**Percentage of peroxisomal fatty acid oxidation in total fatty acid oxidation.** Values are least square means ± SEM (n = 6 for newborn pigs at 0 h and n = 4 for pigs at 24 h). ^*^Significantly different from fatty acid (*P* < 0.05).

Clofibrate and postnatal age had no effect on the distribution of oxidative flux C18:1 and C24:0 between mitochondria and peroxisome (Figure 5, *P* > 0.05). However, the peroxisomal proportion of total fatty acid oxidation was on average 2.3 fold higher from C24:0 (85.5) than C18:1 (37.7%).

### Enzyme activity and mRNA expression

**Figure 6:**
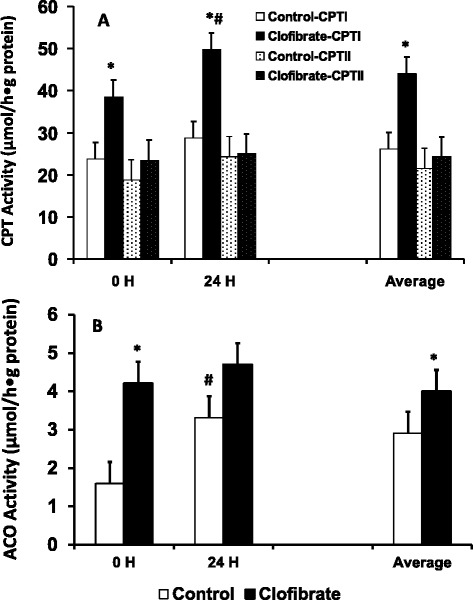
**The effect of maternal clofibrate on enzyme specific activity in newborn pigs at birth and 24 h after delivery by C-section.** Hepatic activity of carnitine palmitoyltransferase (CPT, **A)** and acyl-CoA oxidase (ACO, **B)**. Values are least square means ± SEM (n = 6 for pigs at 0 h and n = 4 for pigs at 24 h). ^*^Significantly different from clofibrate and ^#^ significantly different from postnatal age (*P* < 0.05).

Hepatic CPT specific activity (Figure 6A) measured with and without malonyl-CoA was 69% and 43% higher, respectively, in the clofibrate-exposed pigs (*P* < 0.018). There was no change in the activities with postnatal age (*P* = 0.2). The total and inhibited enzyme activity (μmol/h/g protein) were 31.2, 52.4 on average for the pigs at delivery and 39.3, 64 on average for the 24 h-old pigs. Hepatic ACO activity was increased by 2.3 fold in the clofibrate-exposed pigs (*P* < 0.036; Figure 6B). The enzyme activity also increased by 1.4 fold in 24-h-old pigs, but the increase had no influence on the degree of clofibrate stimulation (*P* > 0.05).

**Figure 7:**
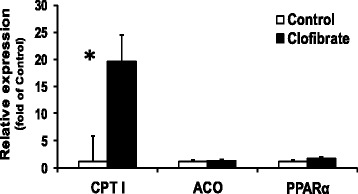
**The effect of maternal clofibrate on mRNA expression in newborn pigs at 0 h. mRNA expression was performed by qtPCR in duplicates.** Values (fold of control) are least square means ± SEM (n = 6). ^*^Significantly different from clofibrate (*P* < 0.05).

The relative mRNA expression of CPT I was increased by 19-fold in liver of the clofibrate-exposed pigs (*P* < 0.0001), but no differences were detected for the relative mRNA abundance of either ACO or PPARα (*P* > 0.05, Figure 7).

## Discussion

Previous research in rodents has shown peroxisome proliferation was observed in the liver of fetuses over 13 days and/or newborns from clofibrate treated dams during gestation [15,23]. The peroxisomal membrane protein 70 and the marker enzymes dihydroxyacetone phosphate acyltransferase and catalase specific activities were significantly increased in the fetal liver of mice at 19 d gestation [15]; indirectly suggesting that clofibrate and/or its metabolites could cross the placenta barrier to enter the circulation system of the fetus in rodent species. However, there was no direct evidence to demonstrate if clofibrate, clofibrate metabolites, or both actually cross the placental barrier. Additionally, the morphology and function of placenta vary greatly among mammals [24]. The type of placenta in swine has been described as a diffuse, folded, and epitheliochorial placenta type that is different from that of rats, which has a discoidal, labyrinthine, and hemotrichorial placenta type [25]. Considering the disparity between the morphology and mechanisms for an extra embryonic membrane attachment in swine, the placental transfer of clofibrate could be different from the rodent species. The aim of this study is to determine if clofibrate or its metabolites traverse porcine placental tissues to evaluate the effect of clofibrate on lipid metabolism in the offspring of pregnant swine fed clofibrate. Our result clearly established that clofibrate is absorbed and hydrolyzed into clofibric acid, and the clofibric acid crosses the porcine placenta with no chemical or structural modifications. The results are also in line with the previous observation that the biofunctional activity of clofibrate is due to clofibric acid [26]. In addition of the analytical results from plasma analyses, the in vitro metabolism measurements strongly support that clofibric acid delivered via the maternal diet entered fetal circulation and induced fetal hepatic fatty acid oxidation. Feeding clofibrate to neonatal pigs for 14 days and to young pigs for 28 days caused a mild increase in the liver weight as a percentage of body weight (3.3%, [11]; 3.8%, [27]). The relative weight of the liver (g/kg body weight) measured in this study showed no difference between the pigs from the clofibrate treated group (26.9 ± 0.09) and control group (27.9 ± 0.15), suggesting that exposure to the pharmacological PPARα agonist clofibrate by sows for 9 days during late pregnancy would not cause a morphological change in the liver of the offspring. Although we did not perform a histological examination, clofibrate has no substantial effect on the peroxisome proliferation in this species at the ages of 7 days and 8 weeks [28]. This is similar to humans but significantly different from the rodent species in which activation of PPARα by clofibrate initiates hepatocyte proliferation and induces the well-known hepatocellular carcinoma [29,30].

Evidence from rodent species demonstrated that maternal supplementation of clofibrate orally or by injection stimulates peroxisomal proliferation, decreases oxygen uptake, and alters lipid metabolism in the liver and intestine of fetuses or neonates. Maternal clofibrate induces cytochrome P4504A mRNA expression [16] and increases ACO and catalase specific activities in the fetal liver and kidneys [31]. Recently, the high relative mRNA expression of PPARα target genes ACO, CPT I, medium- and long-chain acyl-CoA dehydrogenases were observed also in the liver of fetuses from pregnant rats fed with clofibrate and oxidized fat [17]. However, as PPARα is a crucial regulator of lipid metabolism, the role of its activation of target genes in fatty acid oxidative metabolism in the fetal and/or neonatal liver from the pregnant animals receiving clofibrate has not been determined in previous studies. In the current study, we examined both peroxisomal and mitochondrial β-oxidation using ^14^C-labeled long-chain fatty acids C18:1 and C24:0. Results from this study indeed demonstrated that maternal feeding of clofibrate increases hepatic fatty acid oxidation in the pig’s liver at birth and 24 h after birth. The increase appears to be associated with the enhanced CPT I and ACO specific activities in the liver. The results achieved in the newborns exposed to clofibrate prenatally via the maternal diet were similar to the increase in vitro and vivo fatty acid oxidation reported in neonatal pigs receiving clofibrate directly postnatally [10,13]. Because the increase of CPT I specific activity was congruent with the great increase in mRNA expression, the stimulation of fatty acid oxidation in the liver, particularly in the mitochondria, resulted from the gene expression potentially due to the activation of PPARα by clofibrate. In contrast with CPT I, however, the expressions of ACO and PPARα mRNA were not significantly induced in clofibrate-exposed pigs. This suggests that other factors might be involved in the upregulation of ACO specific activity. Data from studies with rodent fetuses indicated that PPARα mRNA expression is associated with the postpartum age and hormonal and/or nutritional status of the mother. The induction of liver PPARα mRNA expression occurs around birth and the expression maintains an elevated level throughout the suckling period [32], while the content of peroxisomes and the activity of peroxisomal enzymes appear to occur in late fetal development and peak dramatically at birth [33]. These results imply that the ACO protein and PPARα mRNA might have reached pinnacle expression at birth. Indeed, the increase in relative PPARα mRNA was not observed in the liver from clofibrate fed rats in a study conducted by Ringseis et al. [34]. They suggested that endogenous free fatty acids might reduce the capability of fibrates to active PPARα, and consequently its metabolic effects, because ACO is the enzyme containing PPAR response element and responds to changes in polyunsaturated fatty acid levels in a PPARα–dependent manner. Furthermore, a different physiological status, such as a fasting state, might provoke a different PPARα response in the liver of fetuses. The induction of peroxisomal oxidation occurs immediately postpartum, is greater in the suckled versus fasted neonatal pigs, and is reliant on the initiation of suckling [35]. Thus, the lower response in mRNA expressions of ACO and PPARα might be due to the developmental stage and the physiological status of the fetuses because the mRNA of PPAR α was also measured in a fasted state in this study.

The effect of the developmental ages of fetuses on oral food intake and gastrointestinal digestion has been described in humans and animals [36,37], but information on the energy metabolism in the fetus is very limited after delivery. Results from earlier studies showed that hepatocytes isolated from term guinea pigs were unable to oxidize fatty acids, but the capability was developed in the first 12 h after birth. The production of ^14^C measured in CO_2_ and ASP from 1-^14^C labeled fatty acids at 6 h was 40–50% of the production at 24 h. At 12 h of age the rate of fatty acid oxidation had already reached the rate at 24 h and did not change during suckling in the first week of life. These data show that the capacity for β-oxidation and ketogenesis develops maximally in this species during the first 6–12 h after birth, and appears to be partly dependent on the development of fatty acid-activating enzymes [38]. Similarly, a low fatty oxidation rate with a high esterification was also observed in hepatocytes isolated from term fetal rabbits, whatever the octanoate concentration in the medium [39]. Consistent with the observations in term guinea pigs and rabbits, the fatty acid oxidation obtained in term pigs at the time of delivery by C-section was also low even when compared with the rate measured in newborn pigs born naturally [5]. The low fatty acid rate was increased significantly in the first 24 h, in which the rate was increased 2.3 fold in mitochondria and 1.9 fold in peroxisomes. However, the increase in mitochondria apparently was not due to an increase of the key enzyme CPT I activity. The activity measured at 24 h was not significantly different from the activity measured at 0 h after the delivery. A similar phenomenon was also observed in newborn and 24 h fasted pigs [5]. The increase might be associated with an increase in the number of mitochondria observed in neonatal pigs during in the first 12 h [40] and/or a decrease of sensitivity of CPT I to malonyl-CoA inhibition observed in 24-h-old pigs [5]. In contrast with mitochondria, the increase of fatty acid oxidation in peroxisomes was accompanied with a 2.3 fold increase of the ACO. Even so, it is notable that the oxidation rate of C24:0 was not significantly promoted during the first 24 h in peroxisomes, suggesting differences between mitochondria and peroxisomes in the capability of oxidizing fatty acids. Because the very long-chain fatty acid exclusively is activated in micorosomes or/and peroxisomes, the low fatty acid oxidation might be associated with a low activity of very long-chain acyl-CoA synthetase. These results imply the impact of postpartum age on the metabolic pathway varied among subcellular compartments.

Peroxisomal fatty acid oxidation catalyzes chain-length shortening of monounsaturated fatty acid and saturated very long-chain fatty acid. Evidence indicated that C24:0 could only be oxidized initially in peroxisomes after activation by the acyl-CoA sythetase enzyme in endoplasmic reticulum and peroxisomes in brain and liver [41,42]. Peroxisomes at least have two enzyme systems for fatty acid activation: 1 for long-chain fatty acid and 2 for very-long-chain fatty acid [42]. Indeed, there was no substantial ^14^C accumulation in either CO_2_ or ASP or both of CO_2_ and ASP from C24:0 in mitochondria. Neither clofibrate-exposure nor age had any influence on the definitely negligible mitochondrial ^14^C accumulation from C24:0 (≤1%). The percentage of C24:0 oxidation in peroxisomes was more than 80% of the total oxidation. Clofibrate had no effect on the relative oxidative capacity of peroxisomes although the efficacy of clofibrate was increased by postpartum age. This result suggests that the very long-chain fatty acid is catabolized exclusively in peroxisomes. In contrast with C24:0, high^14^C accumulations in CO_2_, ASP and CO_2_ + ASP from C18:1 were observed in both mitochondria and peroxisomes, suggesting that C18:1can be oxidized initially in both of the organelles. Similar results were observed also using C18:1 and erucic acid as substrates in our previously study [13].

## Conclusions

In conclusion, clofibrate from maternal oral feeding during gestation is absorbed and hydrolyzed to clofibric acid, which can cross the porcine placenta and enter the fetal circulation system. Fetuses that were exposed to clofibric acid via maternal-placental transfer had a higher hepatic capacity to oxidize fatty acid at birth as compared to control fetuses. The high fatty acid oxidative capacity resulted from an increase in key enzyme activities induced by clofibric acid via activation of PPARα and its target genes. The promoted fatty acid oxidation by activation of PPARα and its target genes was attenuated in fasted newborns with postpartum age. Postpartum age also increased fatty acid oxidation. The increase was associated with development and was not influenced by clofibrate supplementation. Results from this study suggest that clofibrate could be examined as an agent to induce precocious development of ACO and possibly improve milk fat oxidation and pig survivability.

## Abbreviations

ACO: Acyl-CoA oxidase
ASP: Acid soluble products
CPT: Carnitine palmitoyltransferase
C18:1: Oleic acid
C24:0: Lignoceric acid
PPARα: Peroxisomal proliferator-activated receptor
